# siRNAs Targeting Non-Human Species-Specific lncRNAs Trigger Cell Death in Human Colorectal Cancer Cells

**DOI:** 10.7150/jca.99462

**Published:** 2024-09-23

**Authors:** Wan-Ying Feng, Jun-Xiang Zeng, Yan-Ru Chen, Zhe-Ping Fang, Yi Gao, Wei-Jie Zhou

**Affiliations:** 1 Department of General Surgery & Guangdong Provincial Key Laboratory of Precision Medicine for Gastrointestinal Tumor, Nanfang Hospital, Southern Medical University, Guangzhou, China.; 2 Department of Pathology, Guangdong Provincial People's Hospital (Guangdong Academy of Medical Sciences), Southern Medical University, Guangzhou, China.; 3 Department of Research and Teaching, Huizhou Municipal Central Hospital, Huizhou, China.; 4 Department of Hepatobiliary Surgery, Taizhou Hospital of Zhejiang Province, Wenzhou Medical University, Linhai, China.; 5 General Surgery Center, Department of Hepatobiliary Surgery II, Guangdong Provincial, Research Center for Artificial Organ and Tissue Engineering, Guangzhou Clinical Research and Transformation Center for Artificial Liver, Institute of Regenerative Medicine, Zhujiang Hospital, Southern Medical University, Guangzhou, China.; 6 Department of Gastrointestinal and Hernia Surgery, Ganzhou Hospital-Nanfang Hospital, Southern Medical University, Ganzhou, Jiangxi, China.

**Keywords:** siRNA, species-specific lncRNA, colorectal cancer, cell death, dsRNA

## Abstract

Species-specific long non-coding RNAs (lncRNAs) possess numerous unknown functions. We have recently reported that short interfering RNAs (siRNAs) designed to target mouse-specific lncRNAs caused cell death exclusively in human cancer cells, sparing normal human cells and mouse cancer cells. However, it is uncertain whether other non-human species-specific lncRNAs could also be applied as sequential targets for designing anti-tumor therapeutic siRNAs. In this research, we showed that siRNAs targeting rat or zebrafish-specific lncRNAs could exert similar cytotoxic effects against human colorectal cancer (CRC) cells while leaving normal human cells unaffected. Mechanistic investigations revealed that these siRNAs prompted apoptosis or pyroptosis in human CRC cells by triggering an IRF3-independent immune response against exogenous dsRNAs, based on the expression of protein gasdermin E (GSDME). Our study demonstrates that utilizing siRNAs to target non-human species-specific lncRNAs can trigger cell death in human CRC cells, indicating that non-human species-specific lncRNAs could serve as a promising reservoir for target libraries when designing anti-tumor siRNAs.

## Introduction

Long non-coding RNAs (lncRNAs) are transcripts that do not code for proteins and span over 200 nucleotides 1-3. Initially regarded as non-functional genomic compositions 4, their well-regulated expression patterns imply roles beyond mere genomic “noises” 5,6.

Significant progress in high-throughput sequencing and cross-species genomic alignments has led to the extensive identification of non-human lncRNAs 7-10, prompting increased interest in exploring their biological functions. Caren, the lncRNA expressed in the mouse genome, has been demonstrated to contribute to preserving mitochondrial respiratory capacity during pathological conditions by reducing Hint1 expression 11. Another research has revealed that in Drosophila, the lncRNA CRG influenced locomotor activity and climbing ability by positively modulating its neighboring gene CASK 12. The zebrafish lncRNA Cyrano was reported to be involved in the process of brain development by downregulating the expression of MiR-7 13. The distinct expression patterns of lncRNAs associated with development or disease underscore their involvement in critical biological processes across species 14-18.

There are merely low levels of evolutionary constraints imposed on most lncRNAs, with few exhibiting sequence conservations between species while the majority remain species-specific 19-22, although their biological functions remain largely unknown. Recent studies have shown that species-specific RNA processing of lncRNA FAST results in its distinct subcellular distribution, leading to its divergence in biological functions among species 23,24. A study conducted on plants has also revealed the possibility that species-specific lncRNAs might contribute to diversities among species 25. Although various aspects of the biological functions of lncRNAs have been characterized 26-29, most studies concerning species-specific lncRNAs focus on their function within their belonging species, the potential roles of which in other species are still unclear.

We have recently reported that utilizing siRNAs to target mouse-specific lncRNAs could cause death in human cancer cells without affecting normal human cells 30, in this study, we further explore whether siRNAs targeting rat-specific or zebrafish-specific lncRNAs yield similar effects.

## Materials and methods

### Cell maintenance

Human CRC cell lines DLD1 and HCT116, human normal colonic epithelial cell lines NCM460 and FHC, mouse melanoma cell line B16F10, and mouse CRC cell line CT26 were obtained from the American Type Culture Collection. These cell lines were cultured in RPMI 1640 (for HCT116, DLD1, and CT26), DMEM (for NCM460), or DMEM: F12 (for FHC) supplemented with 10% FBS. All cell lines were maintained at 37°C in a 5%CO2 atmosphere and subjected to routine mycoplasma contamination screening using PCR.

### siRNA transfection

siRNAs used in this study were all synthesized by RiboBio (Guangzhou, China), and their sequences are shown in Table S1. For siRNA transfection, the appropriate number of cells were initially seeded in 6-well plates and allowed to incubate overnight. When the cellular confluence reached around 50%, 50nM siRNAs were added along with Lipofectamine 2000 (11668019, Invitrogen). As for treatment with poly(I:C) (P1530-25MG, Sigma-Aldrich), 2ug/mL poly(I:C) was added along with 4μg/mL Lipofectamine 2000 when the confluence reached 80%, and incubated for 16 hours.

### Microscopic imaging

For observation of cellular morphology and death, cells were first seeded in 6-well plates, and bright-field pictures were captured with an Olympus BX53 microscope 48 hours after siRNA transfection. After discarding the cultural supernatant and washing adherent living cells with cold PBS twice, images of living cells were captured. Images were subsequently processed and analyzed using ImageJ software.

### Protein extraction and immunoblot analysis

The cell lysis was performed directly on 6-well plates in lysis buffer (FD9035, FDbio science) supplemented with PMSF (KGP610, KeyGEN BioTECH). Subsequently, the cell lysate was centrifuged at 10000rpm for 5 minutes to collect the supernatant. The concentration of samples was determined with a BCA assay kit (23225, Pierce). Antibodies for detecting caspase-3 (9662S), PARP (9532T), cleaved caspase-3 (9664T), IRF3 (D83B9) and phosphor-IRF3 (D6O1M) were obtained from Cell Signaling Technologies. Other antibodies used in this study included anti-GAPDH (RM2002, Ray), anti-α-tubulin (RM2007, Ray), and anti-GSDME (ab215191, Abcam). Protein bands were eventually visualized using an ECL Chemiluminescence Kit (FD8030, FDbio science).

### Flow cytometric analysis

Both dead and living cells were collected by centrifugation and subsequently washed with precooled PBS twice. Cells were then stained with the Annexin V-FITC/PI Apoptosis Detection Kit (KGA107, KeyGEN BioTECH). Percentages of stained cells would be analyzed with BD LSRFortessa X-20 and the data were processed with FlowJo software. Total percentages of PI- and Annexin-positive cells were used to evaluate the cell death.

### Off-target genes prediction

Off-target genes of siBX323557.1-1/2/3 were predicted as described previously 30. In short, the prediction of siBX323557.1-1/2/3 off-target genes was accomplished according to the complementary matching between the seed regions of siRNAs and mRNAs, similar to how miRNAs match their target genes. Different algorithms were applied in variant databases, and the four databases used in this study were TargetScan 31, miranda 32, RNAhybrid 33, and pita 34.

### Lactate dehydrogenase (LDH) release assay

The supernatant of cells was collected by centrifugation at 250g for 5 minutes. The level of LDH released by cells was assessed using the CytoTox 96 Non-Radioactive Cytotoxicity Assay Kit (G1780, Promega), following the manufacturer's guidelines.

### RNA extraction and quantitative RT-PCR

Centrifugation was initially employed to collect the cells and the RNA extraction was carried out using RNAiso Plus (T9108, Takara). Subsequently, the purity and concentration of samples were determined with the NanoDrop microvolume spectrophotometer (Thermo Fisher). All samples were subjected to reverse transcription into cDNA using Evo M-MLV RTase (AG11605, Accurate Biology) for quantitative analysis. The Quantitative RT-PCR was performed using a QuantStudio 5 real-time PCR instrument (Thermo Fisher), with primer sequences provided in Table S2. The data were processed using the 2^-∆∆Ct^ method, with GAPDH serving as the internal control.

### Statistical analysis

The data were analyzed using either one-way ANOVA or Student's t-test, as appropriate, and presented as mean ± standard deviation (SD). Statistical analysis was conducted using GraphPad Prism software, with significance defined as *p*<0.05.

## Results

### siRNAs targeting mouse-specific lncRNAs trigger cell death in human CRC cells

**Figure 1:**
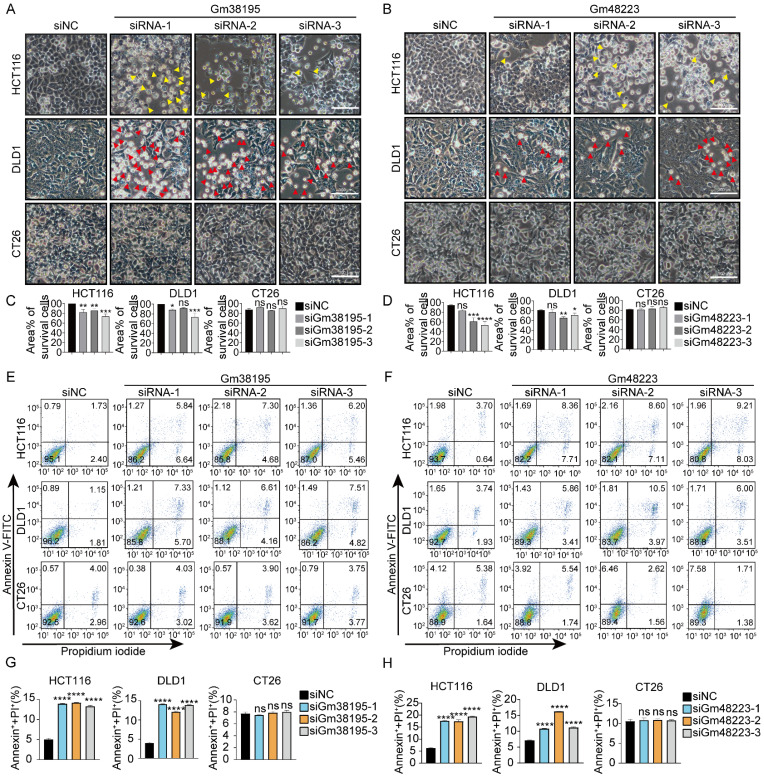
siRNAs designed to target mouse-specific lncRNAs lead to cell death in human CRC cells (**A, B**) Representative microscopic graphs of HCT116, DLD1, and CT26 cells taken 48 hours after transfection with siNC, siRNAs targeting mouse-specific lncRNAs Gm38195 or Gm48223, respectively. Apoptotic and pyroptotic cells were denoted by red or yellow arrowheads, respectively. “siNC” was a 19bp siRNA designed by RiboBio with no targeting sequence, acting as a negative control. “siRNA-1/2/3” meant three 19bp siRNAs targeting different sequences of the lncRNA as indicated. (**C, D**) Percentages of the area covered by living cells under indicated treatments, three independent perspectives were randomly selected for measurement by using Image J. Scale bar, 100μm. (**E -H**) Total cell death was determined by flow cytometry. *p < 0.05, **p < 0.01, ***p < 0.001, ****p < 0.0001, ns, no significant difference.

We have recently reported that utilizing siRNAs to target mouse-specific lncRNAs could cause cell death exclusively in human cancer cells while sparing normal human cells and mouse cancer cells 30. To further confirm the previous findings, 10 more mouse-specific lncRNAs were selected at random (Fig.S1) and 3 different siRNAs were designed for each lncRNA accordingly. 23 out of 30 siRNAs (targeting 10 different mouse-specific lncRNAs) led to lethality in human CRC cell lines DLD1 and/or HCT116. However, none of them caused death in mouse CRC cell line CT26 (Fig. 1, S2). Notably, siRNAs targeting Gm38195, Gm48223, and Gm37091 exhibited greater cytotoxicity against human CRC cells. These results, along with our previous reports, further confirm that siRNAs designed to target mouse-specific lncRNAs can trigger cell death in human CRC cells.

### siRNAs targeting human-specific lncRNAs fail to trigger cell death in mouse cancer cells

**Figure 2:**
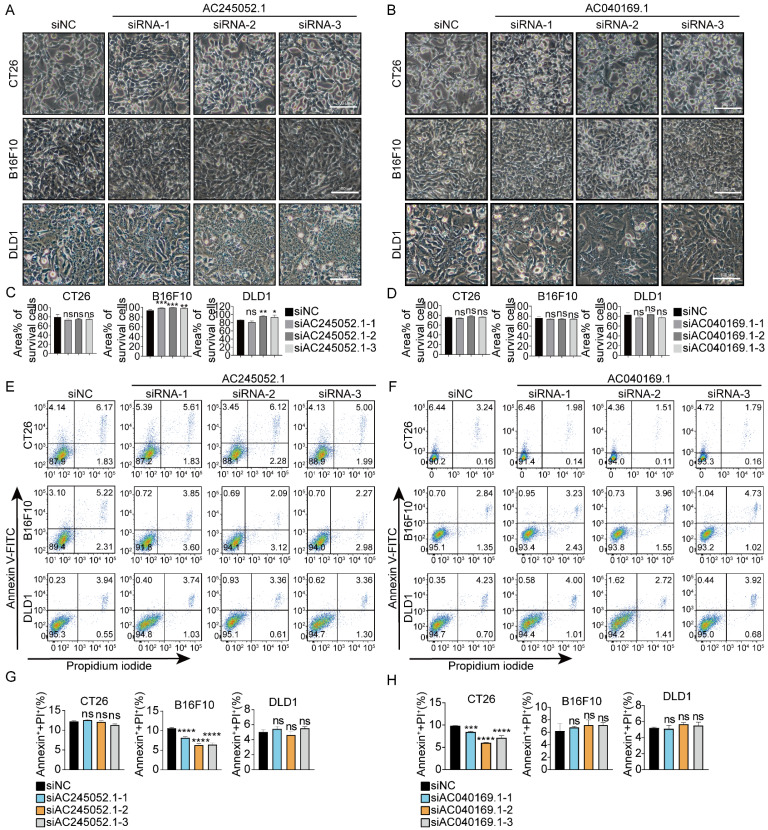
Targeting human-specific lncRNAs with siRNAs fails to trigger cell death in mouse cancer cells (**A, B**) Images representing microscopic views of CT26, B16F10, and DLD1 cells treated with specific siRNAs. (**C, D**) Percentages of the area covered by living cells. Scale bar, 100μm. (**E -H**) Total cell death was assessed by flow cytometry. *p < 0.05, **p < 0.01, ***p < 0.001, ****p < 0.0001, ns, no significant difference.

Next, we explored whether siRNAs targeting human-specific lncRNAs could cause death in mouse cancer cells. 10 human-specific lncRNAs were selected at random (Fig.S3) and 3 siRNAs were designed for each lncRNA accordingly. The results showed that siRNAs targeting human-specific lncRNAs did not cause death in mouse cancer cells CT26 or B16F10, nor did they cause death in human cancer cells DLD1 (Fig. 2, S4).

### siRNAs targeting rat or zebrafish-specific lncRNAs trigger cell death in human CRC cells

**Figure 3:**
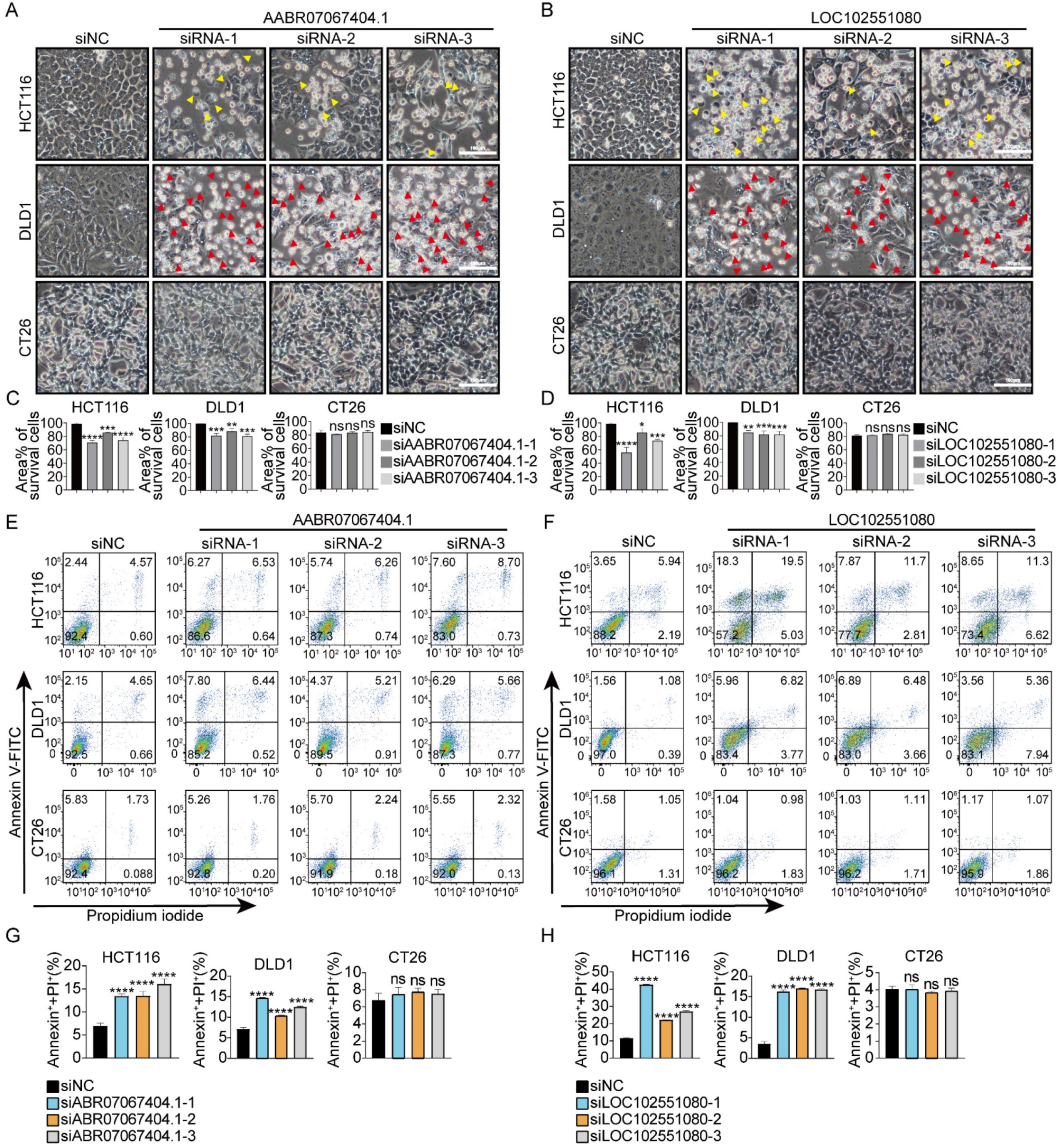
siRNAs targeting rat-specific lncRNAs exhibit specific cytotoxicity against human cancer cells without affecting mouse cancer cells (**A, B**) Representative microscopic pictures of HCT116, DLD1, and CT26 cells under indicated treatments. Apoptotic and pyroptotic cells were denoted by red or yellow arrowheads, respectively. (**C, D**) Percentages of the area covered by living cells. Data were presented in the format of mean ± SD. Scale bar, 100μm. (**E -H**) Percentages of total cell death determined by flow cytometric analysis. *p < 0.05, **p < 0.01, ***p < 0.001, ****p < 0.0001, ns, no significant difference.

**Figure 4:**
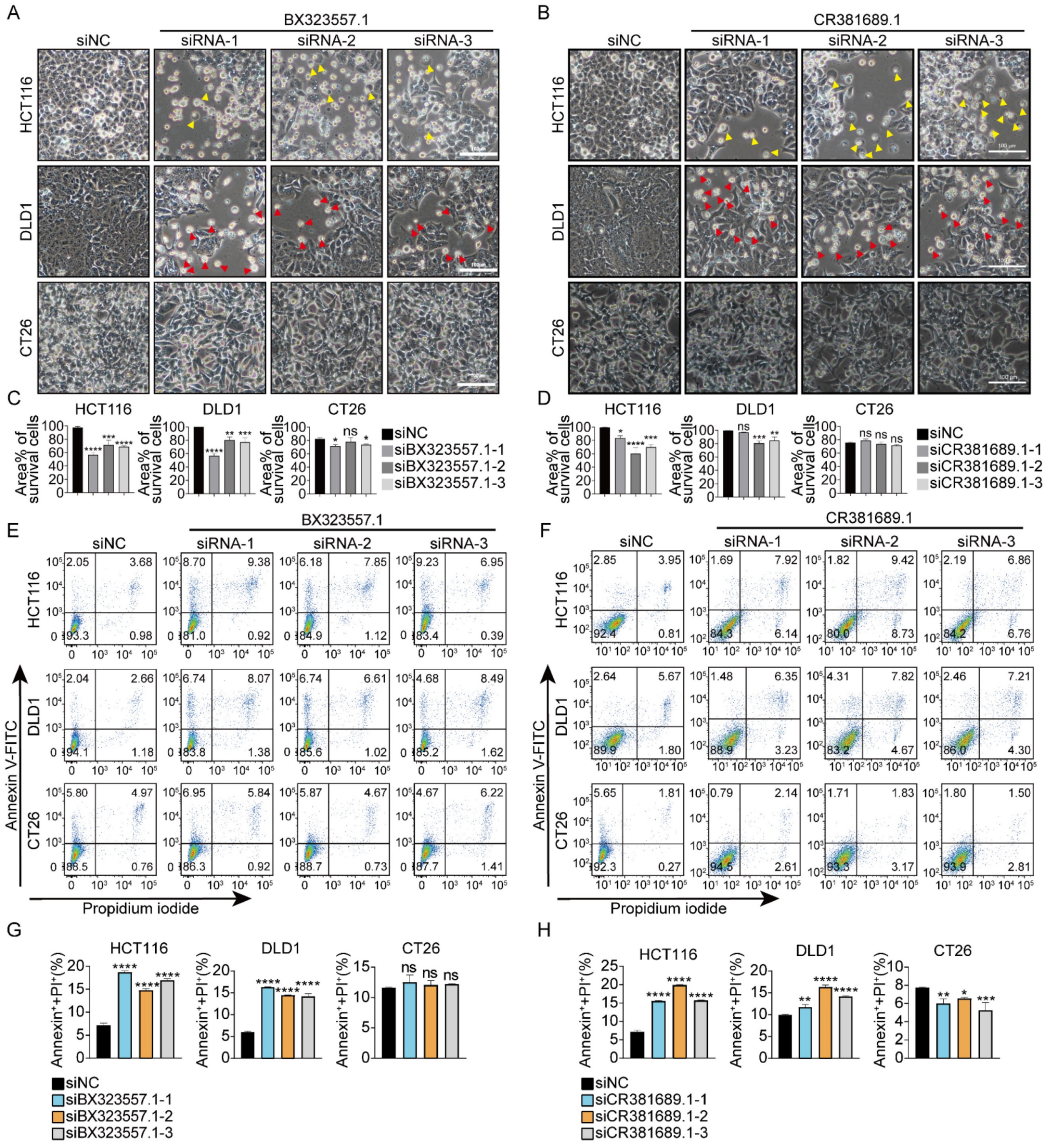
siRNAs targeting zebrafish-specific lncRNAs exhibit specific cytotoxicity against human cancer cells without affecting mouse cancer cells (**A, B**) Representative microscopic pictures of HCT116, DLD1, and CT26 cells treated as indicated. Apoptotic and pyroptotic cells were denoted by red or yellow arrowheads, respectively. (**C, D**) Percentages of the area covered by living cells under specified treatments. Data were presented in the format of mean ± SD. Scale bar, 100μm. (**E -H**) Percentages of total cell death determined by flow cytometric analysis. *p < 0.05, **p < 0.01, ***p < 0.001, ****p < 0.0001, ns, no significant difference.

To determine whether siRNAs designed to target non-human species-specific lncRNAs had a universal cytotoxic effect on human CRC cells, 10 species-specific lncRNAs each from rat and zebrafish were randomly selected (Fig.S5, S6) and three siRNAs were designed for each lncRNA accordingly. 23 of the 30 siRNAs (targeting 10 different rat-specific lncRNAs) triggered cell death in DLD1 and/or HCT116 cells. However, none of them caused death in CT26 cells (Fig. 3, S7). 26 of the 30 siRNAs (targeting 10 different zebrafish-specific lncRNAs) caused death in DLD1 and/or HCT116 cells. However, only 4 of the 30 siRNAs (siRNA-1 and siRNA-3 targeting zebrafish-specific lncRNA BX323557.1, siRNA-1 and siRNA-2 targeting zebrafish-specific lncRNA CABZ01038493.1) slightly prohibited the proliferation of CT26 cells and none of them caused death in CT26 cells (Fig. 4, S8). It should be noted that some siRNAs promoted the proliferation of mouse cancer cells for unknown reasons (Fig.S4, S7, S8).

### siRNAs targeting non-human species-specific lncRNAs prompt apoptosis or pyroptosis in human CRC cells

**Figure 5:**
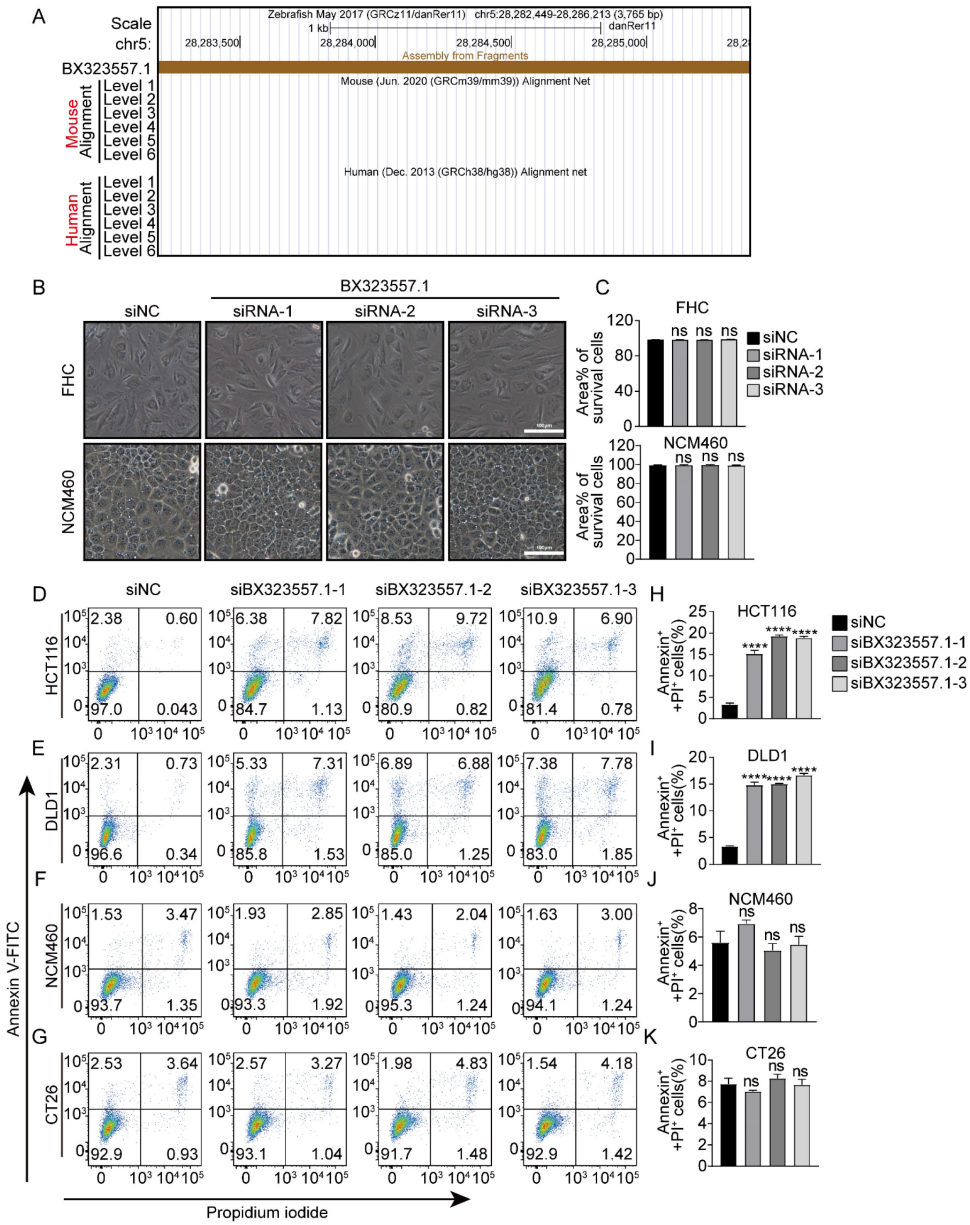
siRNAs targeting zebrafish-specific lncRNA BX323557.1 do not cause death in normal human or mouse cancer cells (**A**) Schematic of genomic alignments of lncRNA BX323557.1 with human and mouse genome through UCSC Genome Browser. (**B**) Representative microscopic pictures of FHC and NCM460 cells treated with the specified siRNAs and (**C**) Percentages of the area covered by living cells. Scale bar, 100μm. (**D -K**) Total cell death was detected by flow cytometry in HCT116, DLD1, NCM460, and CT26 cells treated as indicated. All the data are presented in the format of mean ± SD from three independent experiments. ****p < 0.0001, ns, no significant difference.

To probe the mechanism underlying the cytotoxicity of siRNAs targeting species-specific lncRNAs toward human cancer cells, we selected siRNAs targeting zebrafish-specific lncRNA BX323557.1 (siBX323557.1) for further explorations. We first confirmed that lncRNA BX323557.1 was specifically transcribed in the zebrafish genome, with no ortholog in the human or mouse genome using the UCSC Genome Browser (Fig. 5A). The effect of siBX323557.1 on normal human cells was then investigated in FHC and NCM460 cells, and the results showed that siBX323557.1 did not cause death in either FHC or NCM460 cells (Fig. 5B, C). The total cell death was further confirmed by the flowcytometric analysis, all three siBX323557.1 elevated percentages of PI-/Annexin-positive cells in HCT116 and DLD1 cells, while no significant changes or effects were observed among CT26 and NCM460 cells (Fig. 5D-K).

**Figure 6:**
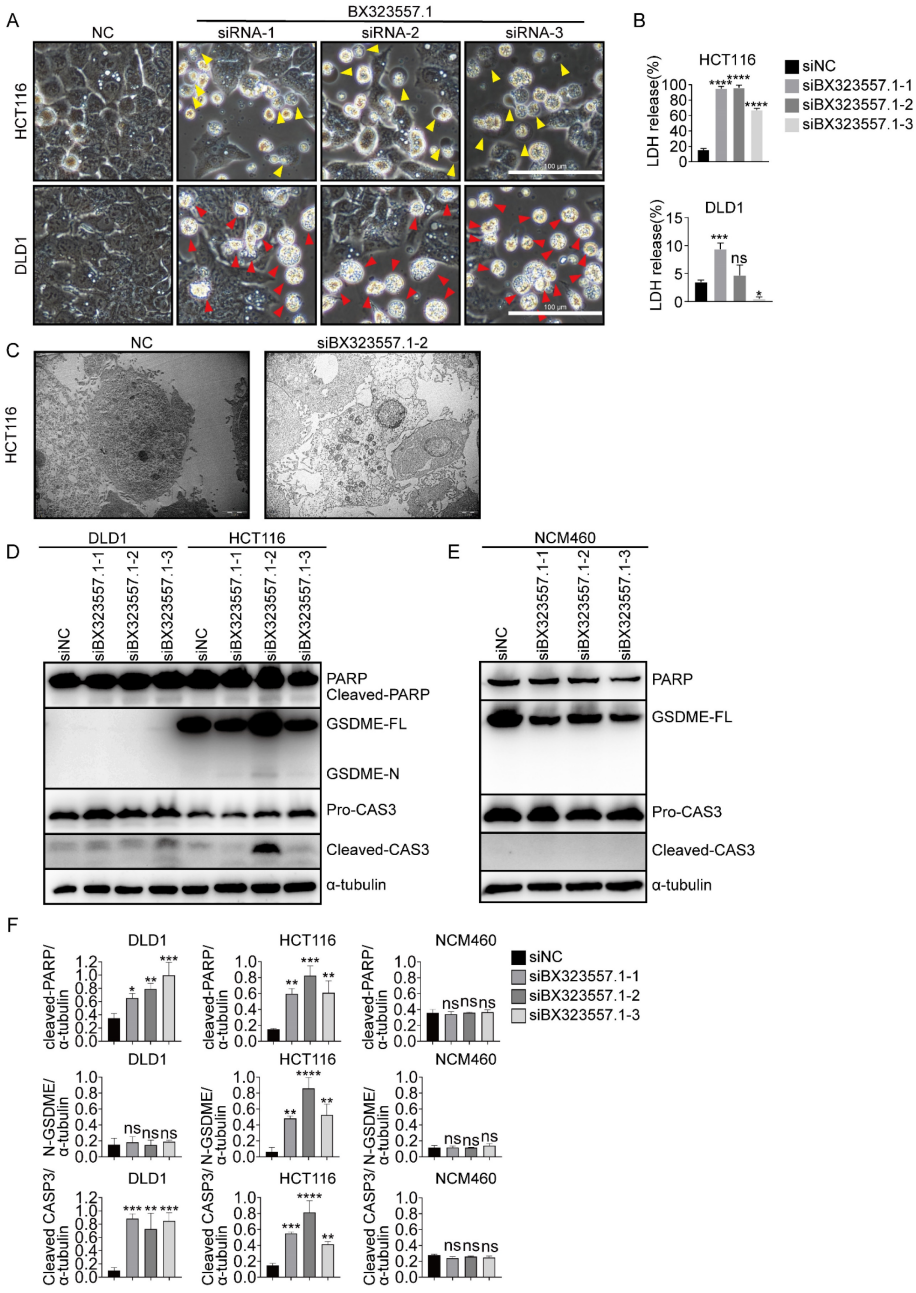
siRNAs designed to target zebrafish-specific lncRNA BX323557.1 prompt apoptosis or pyroptosis in human CRC cells (**A**) Representative microscopic pictures of HCT116 and DLD1 cells treated with siBX323557.1. Apoptotic and pyroptotic cells were denoted by red or yellow arrowheads, respectively. Scale bar, 100μm. (**B**) Comparison of LDH levels in the cultural supernatant between two human CRC cell lines. (**C**) Morphologies observed by the transmission electron microscope of HCT116 cells under indicated treatments. Scale bar, 2μm. (**D, E**) The expression of protein PARP, GSDME, caspase-3, and the corresponding cleaved segments in HCT116, DLD1, and NCM460 cells treated with specified siRNAs. (**F**) Quantified statistics of immunoblotting analyzed by Image J. The level of α-tubulin is applied as an internal control. All the data are presented in the format of mean ± SD from three independent experiments. *p < 0.05, **p < 0.01, ***p < 0.001, ****p < 0.0001, ns, no significant difference.

In addition to different responses between human normal and cancer cells, distinct cellular morphologies could also be observed between two human CRC cell lines. GSDME is expressed in HCT116 cells, while it is absent in DLD1 cells 30. When treated with siRNAs targeting non-human species-specific lncRNAs, pyroptotic bubble formation was mainly observed in GSDME-expressing HCT116 cells, while apoptotic cells were mainly observed in GSDME-absent DLD1 cells (Fig. 1, 3, 4). Clear evidence was provided by the microscopic pictures that following siBX323557.1 treatment, the predominant dying cells of HCT116 exhibited pyroptotic morphologies, such as the formation of large bubbles on the cell membrane and cell swelling. On the other hand, the predominant dying cells of DLD1 agglomerated and shrank, resembling apoptosis (Fig. 6A, C). The LDH content in the cell culture supernatant was evaluated to further confirm the nature of cell death (Fig. 6B). Recent reports showed that N-terminal GSDME due to the cleavage of caspase-3 could execute pyroptosis, thus, the forms of cell death among caspase-3-activated cells were determined by the expression of GSDME 35,36. The immunoblotting results indicated the presence of GSDME in HCT116 cells and its cleavage after transfection with siBX323557.1. However, GSDME was absent in DLD1 cells with or without siRNA stimulation. It should be noted that despite the endogenous expression of GSDME, siBX323557.1 did not prompt apoptosis or pyroptosis in NCM460 cells (Fig. 6D -F).

### siRNAs targeting lncRNA BX323557.1 prompt cell death by triggering an IRF3-independent immune response

**Figure 7:**
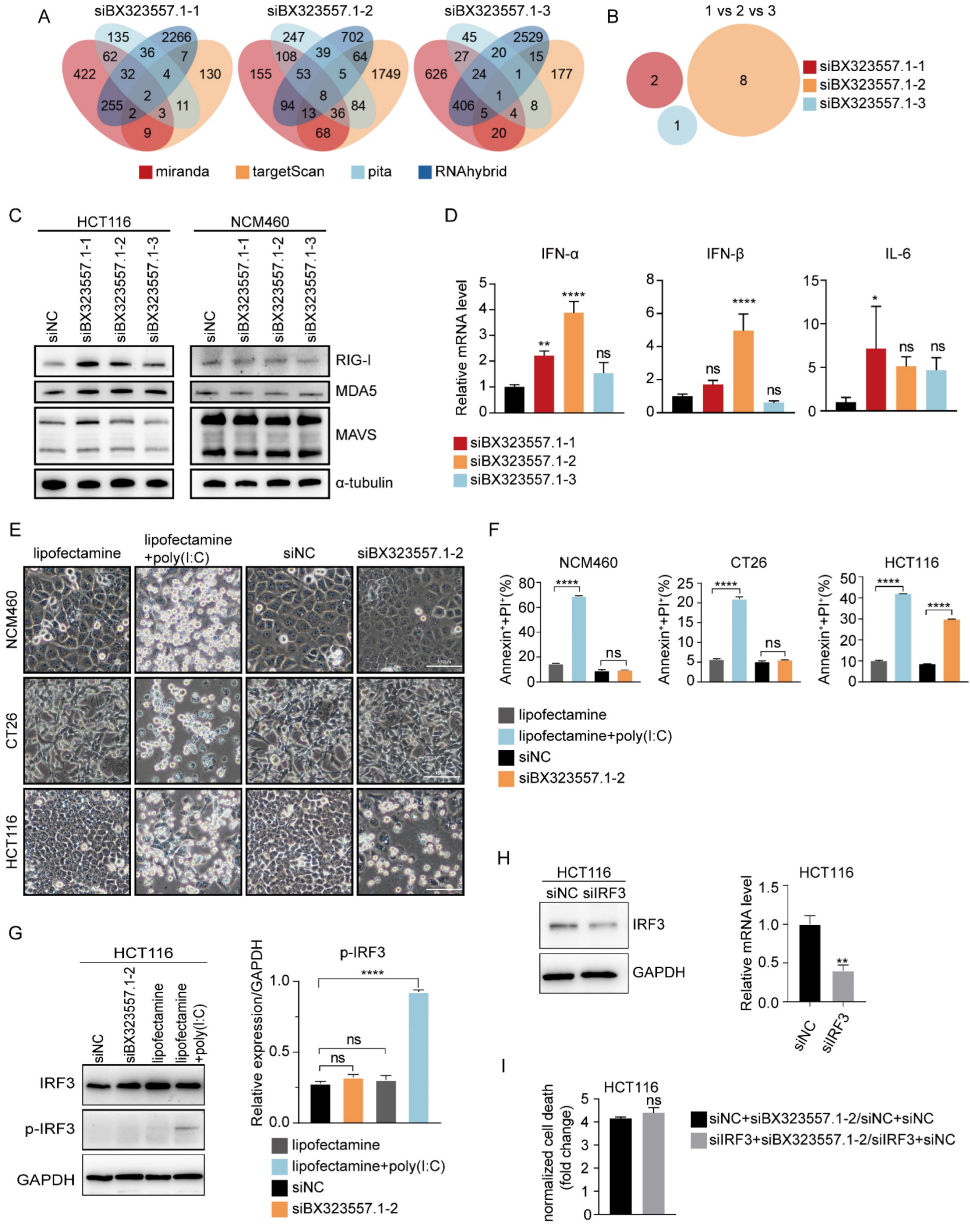
siRNAs targeting zebrafish-specific lncRNA BX323557.1 elicit cell death by triggering an IRF3-independent immune response. (**A**) Off-target genes of siBX323557.1-1/2/3 in the human genome predicted by database pita, miranda, targetScan, and RNAhybrid. (**B**) “1 VS 2 VS 3” referred to the prediction of shared off-target genes of siBX323557.1-1/2/3 in the human genome. (**C**) Expression of dsRNA sensors detected by immunoblotting. (**D**) The relative mRNA levels of type-I interferon genes. (**E**) Representative bright-field pictures of NCM460, CT26, and HCT116 cells under specified treatments. Scale bar, 100μm. (**F**) Total cell death was determined by the flow cytometric analysis. (**G**) The protein expression of IRF3 and p-IRF3 in HCT116 cells upon the indicated treatments. p-IRF3: phosphorylated IRF3. (**H**) The verification of silencing efficiency of siIRF3 by immunoblotting and qRT-PCR. (**I**) The comparison of normalized cell death caused by siBX323557.1-2 with or without IRF3 silencing. *p < 0.05, **p < 0.01, ****p < 0.0001, ns, no significant difference.

As lncRNA BX323557.1 is a zebrafish-specific lncRNA with no ortholog in the human genome, we first need to rule out the prospect that the cytotoxicity of siBX323557.1 against human CRC cells stemmed from the off-target effect. The prediction of off-target genes was carried out by using 4 different databases including TargetScan, RNAhybrid, miranda, and pita. The analysis showed that the three siRNAs targeting lncRNA BX323557.1 did not share any common off-target genes in the human genome, which helped to exclude the possibility of the off-target effect (Fig. 7A, B).

We found that siBX323557.1 upregulated the expression of the cytoplasmic dsRNA sensors MDA5 and RIG-I (Fig. 7C). The expression of type-I interferons was also elevated upon treatment with siBX323557.1 (Fig. 7D). In our previous work, we also found that the cytotoxicity of siRNAs targeting mouse-specific lncRNAs depended on their dsRNA structures 30, suggesting the lethality of siBX323557.1 could be triggering a cellular response against exogenous dsRNAs. To further investigate the previous finding, we compare the cytotoxicity of siBX323557.1-2 with the dsRNA-mimetic poly(I:C), which can activate the RIG-I/MDA5 pathway when administered with lipofectamine, leading to the induction of type I interferon in an IRF3-dependent manner 37-39. The results showed that poly(I:C) could cause death in NCM460 and CT26 cells, to which siBX323557.1 exhibited no significant cytotoxicity (Fig. 7E, F). Immunoblotting results showed that IRF3 was phosphorylated upon treatment with poly(I:C) instead of siBX323557.1 (Fig. 7G). We then knocked down the expression of IRF3 in HCT116 cells with siRNA, whereas total cell death caused by siBX323557.1-2 was not alleviated (Figure 7H, I), suggesting the response triggered by siBX323557.1 was IRF3-independent. These findings indicated that the siBX323557.1 prompted cell death of human CRC cells by triggering an immune response distinct from the one activated by poly(I:C), and may represent a type of new immune response against exogenous dsRNA.

## Discussion

We previously found that 16 out of 23 siRNAs targeting 6 distinct mouse-specific lncRNAs caused death in human cancer cells 30. In this study, 23 out of 30 siRNAs targeting another 10 different mouse-specific lncRNAs were shown to trigger death in human CRC cells. Furthermore, similar cytotoxicity was observed among 23 out of 30 siRNAs targeting 10 different rat-specific lncRNAs and 26 out of 30 siRNAs targeting 10 different zebrafish-specific lncRNAs. However, these siRNAs did not cause death in normal human cells. These results strongly suggest that non-human species-specific lncRNAs could be universally targeted to create anti-tumor therapeutic siRNAs, which unveils a new strategy for designing anti-tumor drugs.

The underlying mechanisms by which these siRNAs work are still unclear. We could rule out the prospect that these siRNAs provoked cell death by targeting common genes due to the off-target effect, not only because sequence analysis showed that they did not target common genes that caused cell death, but also because these siRNAs specifically caused death in cancer cells while leaving normal cells unaffected 40,41. These siRNAs may elicit cell death by triggering an innate immune response against exogenous nucleic acids in host cells, as the expression of cytoplasmic dsRNA sensors and type-I interferons were elevated 42,43. Distinct from the dsRNA-mimetic poly(I:C), the cytotoxicity of siRNAs targeting non-human species-specific lncRNAs exhibits specificity towards human cancer cells and was independent of IRF3, suggesting a novel mechanism. Recent studies have reported that the activation of abnormal proto-oncogenes could regulate the RNA splicing, resulting in a large number of intron-retained mRNAs forming dsRNA structure and accumulating in the cytoplasm of tumor cells, which might trigger an innate immune response and lead to apoptosis 44,45. Further study is needed on detailed molecular and signaling pathways to explain greater sensitivities of human cancer cells shown to the cytotoxic effect of these siRNAs than normal human cells. However, although targeting mouse-specific lncRNAs using siRNAs led to the death of human cancer cells, siRNAs targeting human-specific lncRNAs failed to cause death in mouse cancer cells. In addition, 23 out of 30 siRNAs targeting 10 different rat-specific lncRNAs and 26 of the 30 siRNAs targeting 10 different zebrafish-specific lncRNAs caused cell death in human CRC cells while none of them caused death in mouse cancer cells. These results indicate that human cancer cells possess greater sensitivities to the cell death triggered by these siRNAs than mouse cancer cells. The cytotoxic response of siRNAs targeting exogenous species-specific lncRNAs seems to be a unique property of human cancer cells.

In conclusion, in this study, we selected 10 species-specific lncRNAs each from human, mouse, rat, and zebrafish as templates and designed 3 different siRNAs for each lncRNA. 80% of siRNAs targeting rat, mouse, or zebrafish-specific lncRNA were observed to cause cell death in human CRC cells without affecting normal human cells. However, siRNAs targeting human, rat, or zebrafish-specific lncRNAs did not cause death in mouse cancer cells. These results suggest that human cancer cells possess special sensitivities to cell death caused by siRNAs designed to target non-human species-specific lncRNAs. Therefore, non-human species-specific lncRNAs could be targeted to create anti-tumor therapeutic siRNAs, which unveils a promising strategy for designing anti-tumor drugs.

## Supplementary Material

Supplementary figures and tables.

## Abbreviations

lncRNAs: long non-coding RNAs
siRNAs: short interfering RNAs
CRC: colorectal cancer
GSDME: gasdermin E
LDH: lactate dehydrogenase
SD: standard deviation
