# Supplementary material for: siRNAs Targeting Non-Human Species-Specific lncRNAs Trigger Cell Death in Human Colorectal Cancer Cells
Source: J Cancer. 2024 Sep 23;15(18):5956–67. doi: 10.7150/jca.99462 (PMC11493012; doi:10.7150/jca.99462)
Supplement: Supplementary file 1 — Supplementary figures and tables. [file jcav15p5956s1.pdf]

Supplementary Material

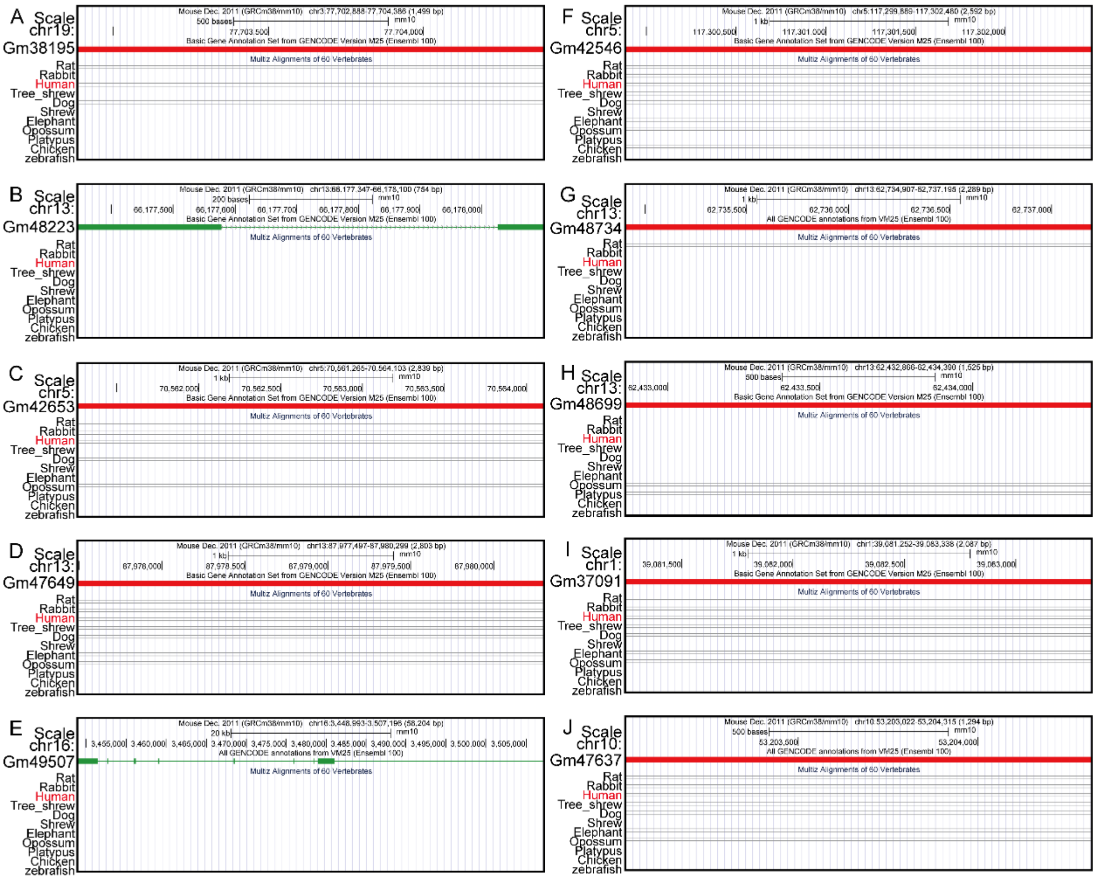

Fig.S1 Genomic alignments of mouse-specific lncRNAs through UCSC genome browser. Schematic of comparative genomic alignments of multiple vertebrates with ten randomly selected mouse-specific lncRNAs including (**A**)Gm38195, (**B**) Gm48223, (**C**) Gm42653, (**D**) Gm47649, (**E**) Gm49507, (**F**) Gm42546, (**G**) Gm48734, (**H**) Gm48699, (**I**) Gm37091, and (**J**) Gm47637. Single line referred to no base in the aligned species, and double line referred that aligning species had one or more unalignable bases in the gap region. These results indicated the absence of orthologs for selected lncRNAs in the human genome.

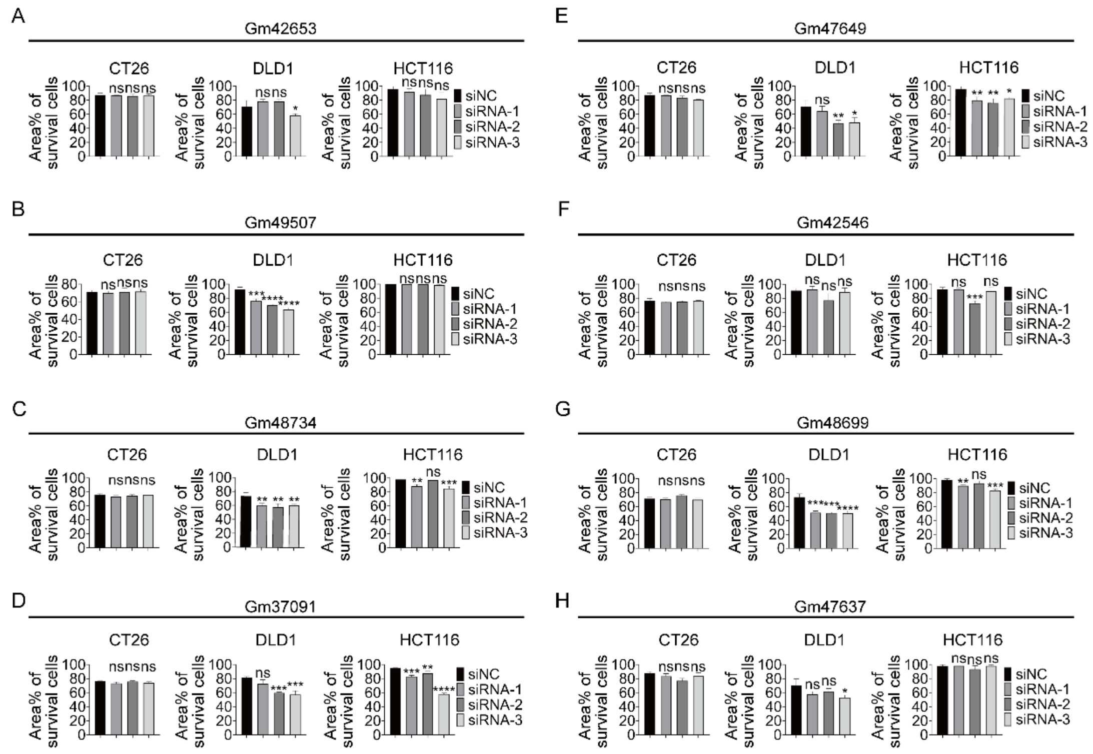

Fig.S2 siRNAs designed to target mouse-specific lncRNAs trigger cell death in human cancer cells specifically but not in mouse cancer cells. (**A -H**) Percentages of the area covered by living cells in CT26, HCT116 and DLD1 cells treated with siRNAs targeting mouse-specific lncRNA Gm42653, Gm49507, Gm48734, Gm37091, Gm47649, Gm42546, Gm48699, and Gm47637, respectively. Three independent perspectives were randomly selected for measurement by using Image J. *$p < 0.05$, **$p < 0.01$, ***$p < 0.001$, ****$p < 0.0001$, ns, no significant difference. Scale bar, 100μm.

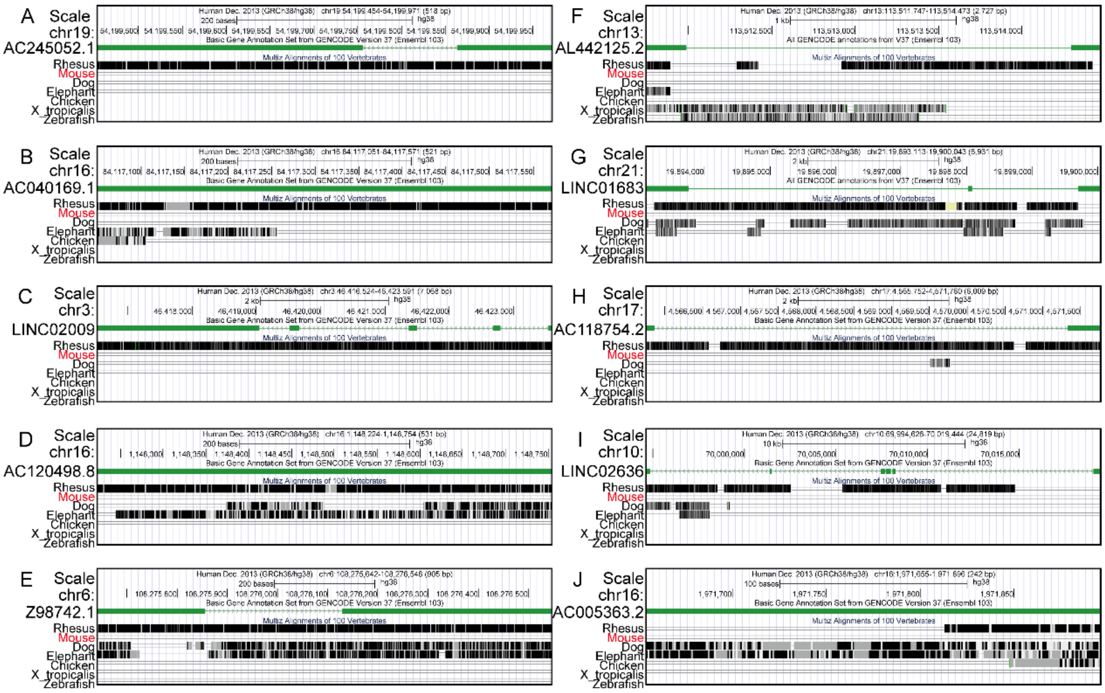

Fig.S3 Genomic alignments of human-specific lncRNAs through UCSC Genome Browser. Schematic of comparative genomic alignments of multiple vertebrates with ten randomly selected human-specific lncRNAs including (**A**) AC245052.1, (**B**) AC040169.1, (**C**) LINC02009, (**D**) AC120498.8, (**E**) Z98742.1, (**F**) AL442125.2, (**G**) LINC01683, (**H**) AC118754.2, (**I**) LINC02636, and (**J**) AC005363.2. These results indicated the absence of orthologs for selected lncRNAs in the mouse genome.

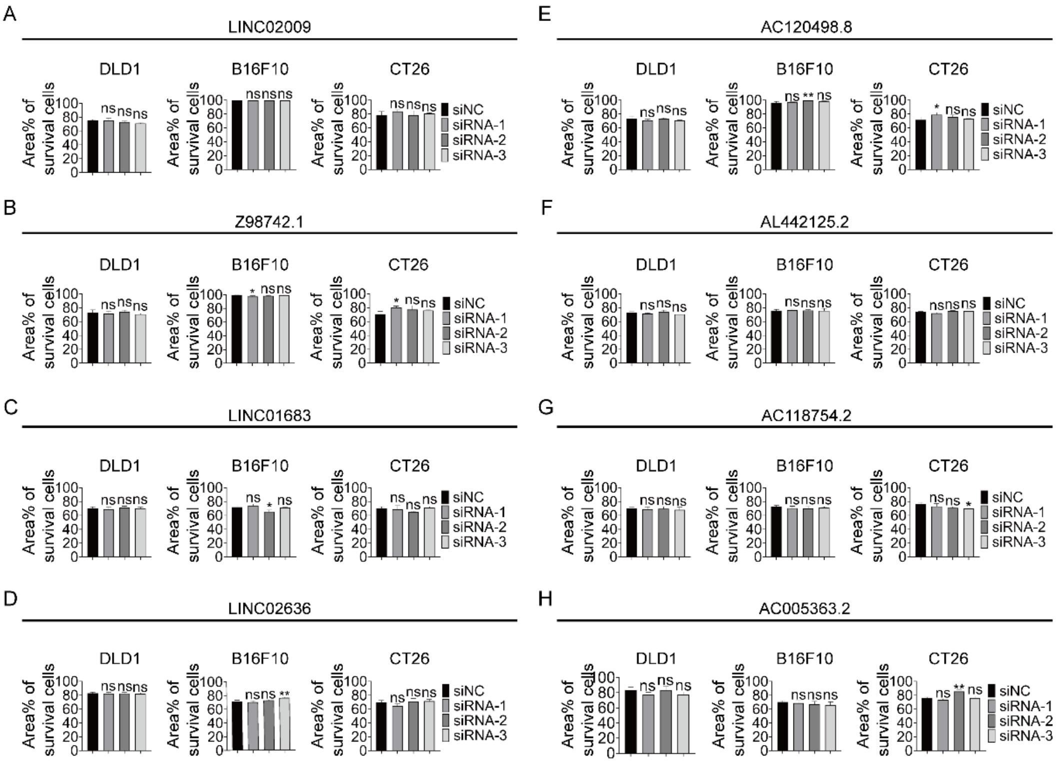

Fig.S4 siRNAs designed to target human-specific lncRNAs fail to trigger cell death in both mouse and human cancer cells. (**A -H**) Percentages of the area covered by living cells of DLD1, B16F10, and CT26 cells treated with siRNAs designed to target human-specific lncRNA LINC02009, Z98742.1, LINC01683, LINC02636, AC120498.8, AL442125.2, AC118754.2, and AC005363.2, respectively. Three independent perspectives were randomly selected for measurement by using Image J. Scale bar, 100μm. **p* < 0.05, ***p* < 0.01, ns, no significant difference.

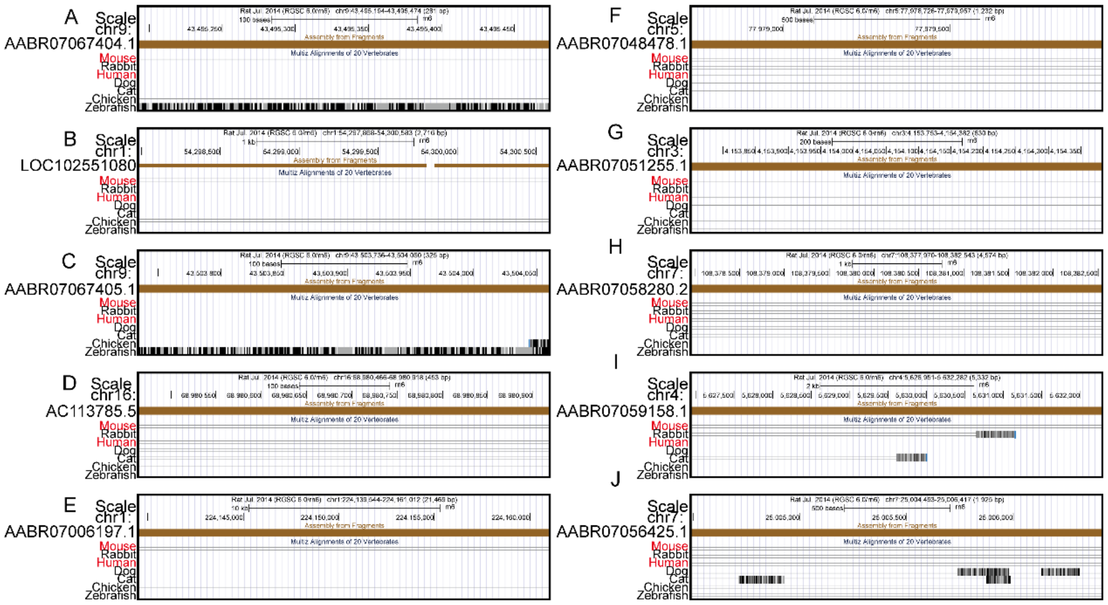

Fig.S5 Genomic alignments of rat-specific lncRNAs through UCSC Genome Browser. Schematic of comparative genomic alignments of multiple vertebrates with ten randomly selected rat-specific lncRNAs including (**A**) AABR07067404.1, (**B**) LOC102551080, (**C**) AABR07067405.1, (**D**) AC113785.5, (**E**) AABR07006197.1, (**F**) AABR07048478.1, (**G**) AABR07051255.1, (**H**) AABR07058280.2, (**I**) AABR07059158.1, and (**J**) AABR07056425.1. These results indicated the absence of orthologs for the selected lncRNAs in both of the mouse and human genome.

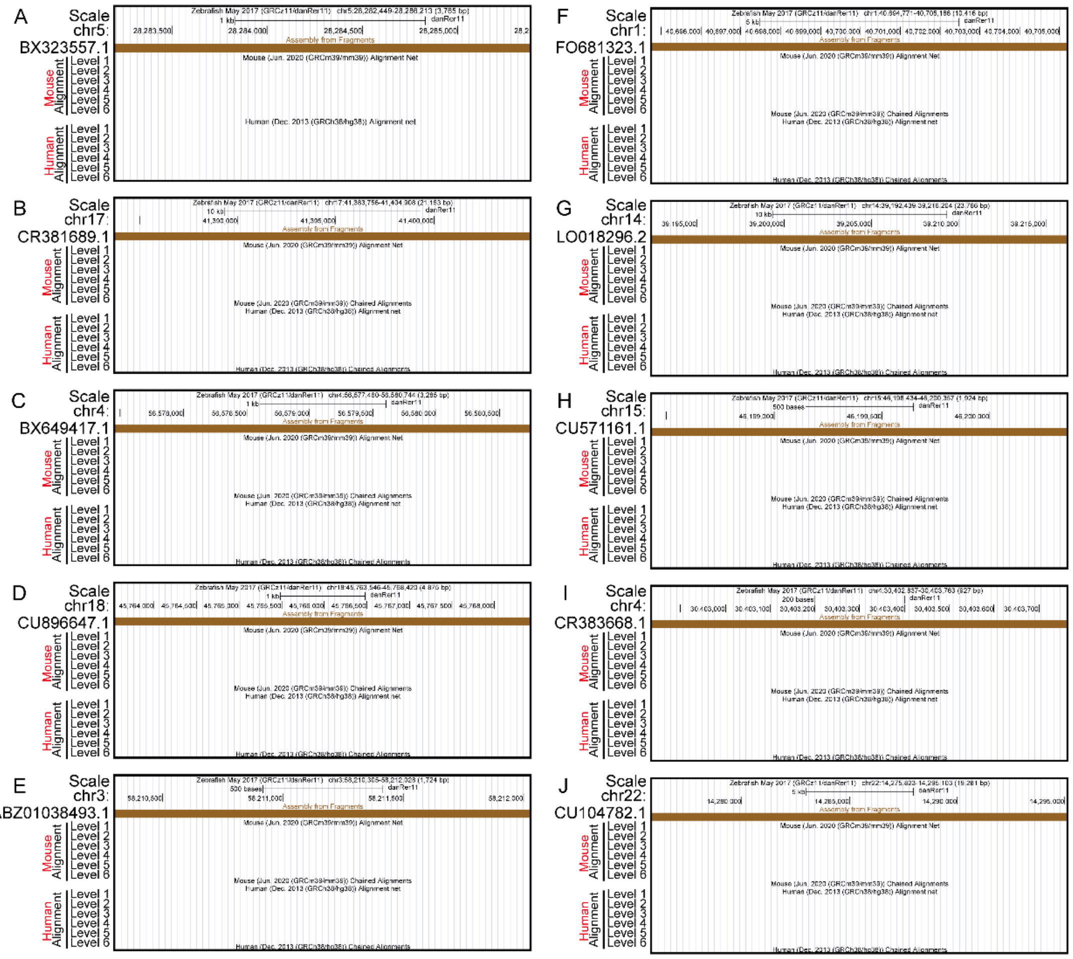

Fig.S6 Genomic alignments of zebrafish-specific lncRNAs through UCSC Genome Browser. Schematic of comparative genomic alignments of both human and mouse genome with nine randomly selected zebrafish-specific lncRNAs including (**A**) BX323557.1, (**B**) CR381689.1, (**C**) BX649417.1, (**D**) CU896647.1, (**E**) CABZ01038493.1, (**F**) FO681323.1, (**G**) LO018296.2, (**H**) CU571161.1, (**I**) CR383668.1, and (**J**) CU104782.1. These results indicated the absence of orthologs for the selected lncRNAs in both of the mouse and human genome.

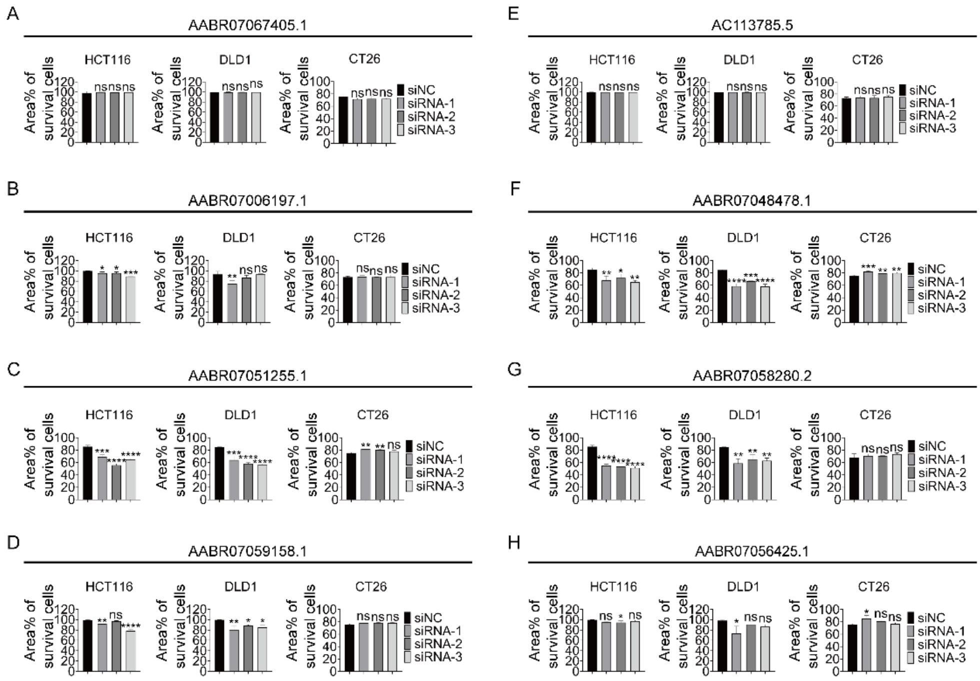

Fig.S7 Utilizing siRNAs to target rat-specific lncRNAs trigger cell death specifically in human cancer cells but not in mouse cancer cells. (**A -H**) Percentages of the area covered by living cells in HCT116, DLD1 and CT26 cells treated with siRNAs designed to target rat-specific lncRNA AABR07067405.1, AABR07006197.1, AABR07051255.1, AABR07059158.1, AC113785.5, AABR07048478.1, AABR07058280.2, and AABR07056425.1, respectively. Three independent perspectives were randomly selected for measurement by using Image J. Scale bar, 100μm. *$p < 0.05$, **$p < 0.01$, ***$p < 0.001$, ****$p < 0.0001$, ns, no significant difference.

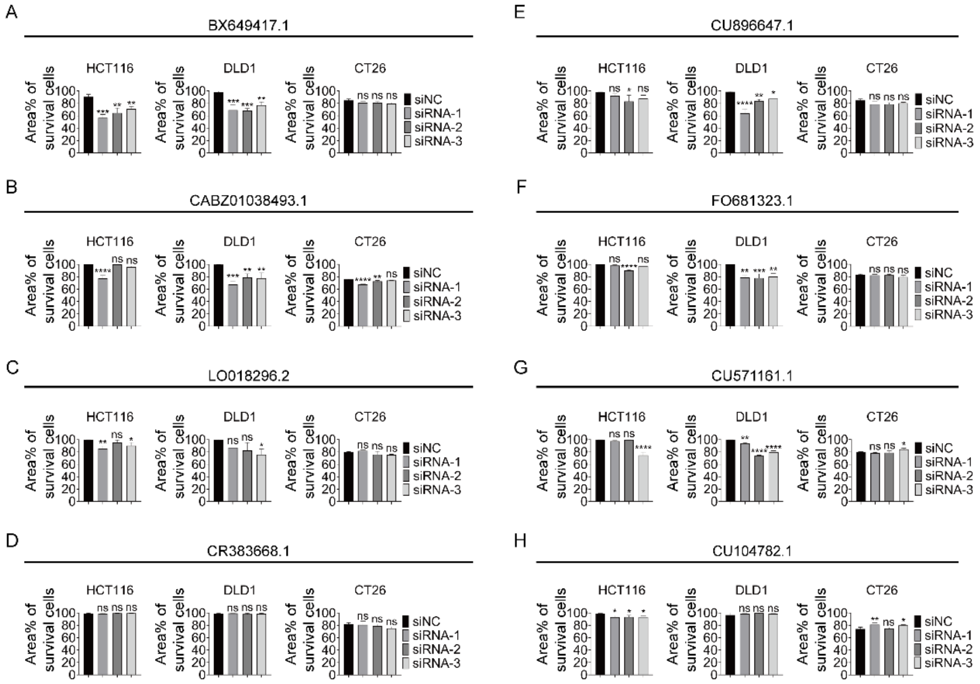

Fig.S8 Utilizing siRNAs to target zebrafish-specific lncRNAs trigger cell death specifically in human cancer cells but not in mouse cancer cells. (**A -H**) Percentages of the area covered by living cells in HCT116, DLD1 and CT26 cells treated with siRNAs designed to target zebrafish-specific lncRNA BX649417.1, CABZ01038493.1, LO018296.2, CR383668.1, CU896647.1, FO681323.1, CU571161.1, and CU104782.1, respectively. Three independent perspectives were randomly selected for measurement by using Image J. Scale bar, 100μm. *$p < 0.05$, **$p < 0.01$, ***$p < 0.001$, ****$p < 0.0001$, ns, no significant difference.

Table S1. Sequences of siRNAs

Name	Sequence
**siRNAs designed to target mouse-specific lncRNAs**	
si-Gm38195-1	Sense:5'-CGUUAAGCAACAACAACAAdTdT-3'
si-Gm38195-2	Sense:5'-GUGAAAGGAUGCACCUAAUdTdT-3'
si-Gm38195-3	Sense:5'-CACAUAAGCAACUCUCAUAdTdT-3'
si-Gm49507-1	Sense:5'-CAACAUCUGGUGCCCACAUdTdT-3'
si-Gm49507-2	Sense:5'-GACAAACCCUGCAGAAGUUdTdT-3'
si-Gm49507-3	Sense:5'-GUGACCUGAAGGUAUUGAAdTdT-3'
si-Gm47649-1	Sense:5'-CCACUACUGAUUCAUGCAAdTdT-3'
si-Gm47649-2	Sense:5'-CCUGGUAUCAUUCUAGGAAdTdT-3'
si-Gm47649-3	Sense:5'-GUGUUGAAGUUUCCCACUAdTdT-3'
si-Gm42653-1	Sense:5'-GGAGUAACUAUUAGAAGCAdTdT-3'
si-Gm42653-2	Sense:5'-GGAUACAGGAGCAGAUAAAdTdT-3'
si-Gm42653-3	Sense:5'-CUGUAAAGGCAGACAAGAAdTdT-3'
si-Gm47637-1	Sense:5'-GCAUCAACAACCUCUCUGAdTdT-3'
si-Gm47637-2	Sense:5'-CCACUAUCCUCUAUACAAAdTdT-3'
si-Gm47637-3	Sense:5'-CCCAAUAUCCUGCUUCUAUdTdT-3'
si-Gm48699-1	Sense:5'-CCAUCAUGACCUCGAUCAAdTdT-3'
si-Gm48699-2	Sense:5'-GCGACCCUACAAUAAAGAAdTdT-3'
si-Gm48699-3	Sense:5'-CCAGAUUAUUGGGAACAUAdTdT-3'
si-Gm48734-1	Sense:5'-GCACUUCUGCUACAUAUGUdTdT-3'
si-Gm48734-2	Sense:5'-ACCUCUCUUUCUUUAAGCUdTdT-3'
si-Gm48734-3	Sense:5'-GCUAUUCCACUAGCUAUUAdTdT-3'
si-Gm42546-1	Sense:5'-CAAGGUGAAUGCAAAUCUAdTdT-3'
si-Gm42546-2	Sense:5'-GCUCUUAGGUCAUGUAACAdTdT-3'
si-Gm42546-3	Sense:5'-GACCUAGUCCAGGUAUAUAdTdT-3'
si-Gm48223-1	Sense:5'-CAAGGAAUGGCUAAUAACUdTdT-3'
si-Gm48223-2	Sense:5'-GGUACCCACAAAGCAACUUdTdT-3'
si-Gm48223-3	Sense:5'-CCAUGCAUCUCUCCAUUCAdTdT-3'
si-Gm37091-1	Sense:5'-GACCUAGUCCAGGUAUAUAdTdT-3'
si-Gm37091-2	Sense:5'-GGCCACCACUUACUAGAUAdTdT-3'
si-Gm37091-3	Sense:5'-GGCUCUGAAAUAGCUUAUUdTdT-3'
**siRNAs designed to target human-specific lncRNAs**	
si-LINC02009-1	Sense:5'-GAACCUUACACCCACAUAAdTdT-3'
si-LINC02009-2	Sense:5'-UGGAGCAUUACCUGUCAUUdTdT-3'
si-LINC02009-3	Sense:5'-GAGCAGUAGUUUCCCUAUAdTdT-3'
si-Z98742.1-1	Sense:5'-CAGAGACUACACUUUCCAUdTdT-3'
si-Z98742.1-2	Sense:5'-GGAUCAGGGCAGAAAUAAAdTdT-3'
si-Z98742.1-3	Sense:5'-GGAUACAAUGUCUGAAAGAdTdT-3'
si-AC245052.1-1	Sense:5'-GUCACUAUCUCCUCAGGAAdTdT-3'
si-AC245052.1-2	Sense:5'-CAGCGUCCUCCUCUGUAAAdTdT-3'
si-AC245052.1-3	Sense:5'-GAUUACAGCUCGUGUUCUUdTdT-3'

si-AC120498.8-1	Sense:5'-AGACGCAGCAGCAAUGAUUdTdT-3'
si-AC120498.8-2	Sense:5'-GAGUGCUUGAAGGAUCACUdTdT-3'
si-AC120498.8-3	Sense:5'-GCCUUAAAUUUCCUUCUCUdTdT-3'
si-LINC01683-1	Sense:5'-CGAUAUUAAGCACCAUACUdTdT-3'
si-LINC01683-2	Sense:5'-GCUUCUUAUACUCCACAUAdTdT-3'
si-LINC01683-3	Sense:5'-GCAAUCCAAAGUGGACCAAdTdT-3'
si-LINC02636-1	Sense:5'-CCAGCUUGCUUUGGAACUAdTdT-3'
si-LINC02636-2	Sense:5'-CAAUGCUGCGAUGAAUACAdTdT-3'
si-LINC02636-3	Sense:5'-GAAUACACCUGUGCAUUCUdTdT-3'
si-AC118754.2-1	Sense:5'-CGAAUGACAACAUGCUAUAdTdT-3'
si-AC118754.2-2	Sense:5'-CUGGUGACUAACUGGUUUAdTdT-3'
si-AC118754.2-3	Sense:5'-CUUCGAGAUAUCAUCUGUAdTdT-3'
si-AL442125.2-1	Sense:5'-GCAGAAUAUCUGAGCAAAUdTdT-3'
si-AL442125.2-2	Sense:5'-GUAUCAAGAACGUCUGUAAdTdT-3'
si-AL442125.2-3	Sense:5'-CGUGAACUGCCCUUAUGGAdTdT-3'
si-AC040169.1-1	Sense:5'-GCCAGGAUCUAGAAAGACUdTdT-3'
si-AC040169.1-2	Sense:5'-GGCGGUAGCUCGUUAAGAUdTdT-3'
si-AC040169.1-3	Sense:5'-GUGCGAAACGGAGGACCUUdTdT-3'
si-AC005363.2-1	Sense:5'-GGCUCUUUGAUACCAGUAUdTdT-3'
si-AC005363.2-2	Sense:5'-CCGUGCGUAGAGCCGUCAAdTdT-3'
si-AC005363.2-3	Sense:5'-GGCUCUUGAUAAAUAACGUdTdT-3'
**siRNAs designed to target rat-specific lncRNAs**	
si-AABR07051255.1-1	Sense:5'-GUGAGUCCUUGACCUGUGAdTdT-3'
si-AABR07051255.1-2	Sense:5'-ACAGCCUGUGAGUCCUUGAdTdT-3'
si-AABR07051255.1-3	Sense:5'-UCCUUGACCUGUGAUGGCUdTdT-3'
si-LOC102551080-1	Sense:5'-GUCACAGUGUGACCCAGAAdTdT-3'
si-LOC102551080-2	Sense:5'-GAGGCCUACCUGGAAAGUUdTdT-3'
si-LOC102551080-3	Sense:5'-CCUUGACAAGUUUCCUGCAdTdT-3'
si-AABR07067404.1-1	Sense:5'-CUGUGCUAUGCCUCUACAUdTdT-3'
si-AABR07067404.1-2	Sense:5'-CUACUGCGUAUCCUCUAUAdTdT-3'
si-AABR07067404.1-3	Sense:5'-UGUAUGUUCUCUACAUCUAdTdT-3'
si-AABR07059158.1-1	Sense:5'-GGUCACUCAUGGCCAUAAUdTdT-3'
si-AABR07059158.1-2	Sense:5'-CCAGAGCAUUAGCUUAGGAdTdT-3'
si-AABR07059158.1-3	Sense:5'-CUGGAGUAACAGAGACUCAdTdT-3'
si-AABR07056425.1-1	Sense:5'-GGUGGUUACUACUGAACAAdTdT-3'
si-AABR07056425.1-2	Sense:5'-GGACCUUCGUAACGGAAAAdTdT-3'
si-AABR07056425.1-3	Sense:5'-CAAGACUGGCAUGGACCUUdTdT-3'
si-AABR07067405.1-1	Sense:5'-GGAUACGCAGAGAUGUAGAdTdT-3'
si-AABR07067405.1-2	Sense:5'-GAGAGAUGUAGAGGAGAUAdTdT-3'
si-AABR07067405.1-3	Sense:5'-GCAGUAGAUGUAGAGGAUAdTdT-3'
si-AABR07058280.2-1	Sense:5'-UGAGAGUCUUACAUAUGGAdTdT-3'
si-AABR07058280.2-2	Sense:5'-UCAGGGAUCACAAUCUAUAdTdT-3'
si-AABR07058280.2-3	Sense:5'-GAGACUACAGCUACACUGAdTdT-3'

si-AC113785.5-1	Sense:5'-GUUGGGAGACCUUUAAUGAdTdT-3'
si-AC113785.5-2	Sense:5'-GAGUAAUUGUGGCUUCGUAdTdT-3'
si-AC113785.5-3	Sense:5'-GUCCAUUUCCUGAAGAUUUdTdT-3'
si-AABR07006197.1-1	Sense:5'-ACAUCCAUAUACCUGGCGAdTdT-3'
si-AABR07006197.1-2	Sense:5'-GGAAAUUUGAAGUGUUCGUdTdT-3'
si-AABR07006197.1-3	Sense:5'-GAAUUCGGAGAGAGGGUUAdTdT-3'
si-AABR07048478.1-1	Sense:5'-CCCAUGGUUCUCUCACUAUdTdT-3'
si-AABR07048478.1-2	Sense:5'-CUAUCUCCCACCUUAAUCUdTdT-3'
si-AABR07048478.1-3	Sense:5'-CCACCUUAAUCUCUGCCCAdTdT-3'
**siRNAs designed to target zebrafish-specific lncRNAs**	
si-BX323557.1-1	Sense:5'-CGUCAGUAAUGUCAAACAAdTdT-3'
si-BX323557.1-2	Sense:5'-CACGCCAUUUACCGCCAAAdTdT-3'
si-BX323557.1-3	Sense:5'-UGAAUUCACAUCCACAAAAdTdT-3'
si-CR383668.1-1	Sense:5'-GGACGUAGCUAGCAAAAGUdTdT-3'
si-CR383668.1-2	Sense:5'-CUGGUGUUUCACUCUCCUAdTdT-3'
si-CR383668.1-3	Sense:5'-CCUAAGGUGUAAGCUAUAAdTdT-3'
si-CU571161.1-1	Sense:5'-AGUGCAUCCAGGUGGUUAUdTdT-3'
si-CU571161.1-2	Sense:5'-CUGUCAGCUUUGCAUUUAAdTdT-3'
si-CU571161.1-3	Sense:5'-GCAGAAUAAAACCAUUCAGdTdT-3'
si-CU104782.1-1	Sense:5'-CAUCCUGAAGACGAAUGAAdTdT-3'
si-CU104782.1-2	Sense:5'-GCAUUACAAGCGGAUAUAAdTdT-3'
si-CU104782.1-3	Sense:5'-GCAACAACAACUGCAAACUdTdT-3'
si-BX649417.1-1	Sense:5'-GGCAAUGAAGAGUCCUAUAdTdT-3'
si-BX649417.1-2	Sense:5'-CAACCAGUCUUCACGACAUdTdT-3'
si-BX649417.1-3	Sense:5'-GCUGUAAGCUCUAUUCAAAdTdT-3'
si-CU896647.1-1	Sense:5'-CAUUACGUGUGCAUGACGUdTdT-3'
si-CU896647.1-2	Sense:5'-UGCAUGACGUGUGGCCAUUdTdT-3'
si-CU896647.1-3	Sense:5'-CGAUCAUUACUCUAACGUUdTdT-3'
si-CR381689.1-1	Sense:5'-CUACGUCAGGGAAGGAGAAdTdT-3'
si-CR381689.1-2	Sense:5'-CGUCAGGGAAGGAGAAAGAdTdT-3'
si-CR381689.1-3	Sense:5'-GGAGAAAGAAUUGCAUGAAdTdT-3'
si-CABZ01038493.1-1	Sense:5'-GUCAUCUCAAGCCAACAAUdTdT-3'
si-CABZ01038493.1-2	Sense:5'-AUGUAUCAGCCUCCUUAUAdTdT-3'
si-CABZ01038493.1-3	Sense:5'-GCAACAACUCAAUGCAACAdTdT-3'
si-FO681323.1-1	Sense:5'-CACAUUUAGACUCUGCAAAdTdT-3'
si-FO681323.1-2	Sense:5'-GCUAGAAUCUUCCAUUUCAdTdT-3'
si-FO681323.1-3	Sense:5'-CAUCCAAUUUACUUACAGUdTdT-3'
si-LO018296.2-1	Sense:5'-UGUUGAGUUGCUUCGUUAAdTdT-3'
si-LO018296.2-2	Sense:5'-CUACAUUGGGCAUUUAUAAdTdT-3'
si-LO018296.2-3	Sense:5'-GUCAUCUUAUGGUGAUAGAdTdT-3'

Table S2. Primer pairs of candidate genes for real-time PCR.

Gene	Forward primer (5’-3’)	Reverse primer (5’-3’)

IFN-α	GCCTCGCCCTTTGCTTTACT	CTGTGGGTCTCAGGGAGATCA
IFN-β	ATGACCAACAAGTGTCTCCTCC	GGAATCCAAGCAAGTTGTAGCTC
IL-6	ACTCACCTCTTCAGAACGAATTG	CCATCTTTGGAAGGTTCAGGTTG
GAPDH	GACTCATGACCACAGTCCATGC	AGAGGCAGGGATGATGTTCTG
